# Understanding the impact of fertility history on health outcomes in later life.

**DOI:** 10.23889/ijpds.v7i3.2061

**Published:** 2022-08-25

**Authors:** Lee Williamson, Chris Dibben

**Affiliations:** 1 University of Edinburgh

## Objectives

Aims of this research, involving data linkage and health outcomes, is to gain a full understanding of the impact of both fertility histories and childlessness on health outcomes mid-life accounting for socio-economic background and area of residence. The research draws on and extends work on reproductive histories and life-course outcomes.

## Approach

We aim to extend this area of research, specifically for Scotland, using Census data (1991-2011) from the Scottish Longitudinal Study (SLS) linked to health data. The Census health measures – including the 2011 Census health condition question on mental health - are the research outcomes and the explanatory information is from Census socio-economic data (captured around peak fertility for the research cohort in 1991), along with the SMR02 Maternity and SMR04 Mental Health datasets. The time-frame for available data allows 20 year follow-up from the 1991 Census to mid-life for specific female SLS birth cohorts (born 1959-1966, aged ~45-52 in 2011).

## Results

From preliminary modelling we initially find, for this specific female research cohort, high birth parity to be an important factor in relation to self-reported mental health conditions at follow-up in 2011, but not once socio-economic and area-level variables are controlled for.

## Conclusion

Preliminary modelling also highlights that relationship status – single, married or cohabiting – to be important over that of legal marital status as recorded at Census. For limiting long-term illness as a health outcome the findings are similar.

